# The Positive and Negative Impacts of Researching Emotionally Challenging Topics

**DOI:** 10.1007/s10805-026-09747-y

**Published:** 2026-06-13

**Authors:** Kristine Brance, Tina N. Skinner, Sarah L Halligan, Heather Girling, Emily Tsang

**Affiliations:** 1 Queen’s University Belfast, Belfast, UK; 2 Department of Social and Policy Sciences, University of Bath, Bath, UK; 3 Department of Psychology, University of Bath, Bath, UK; 4 Safety, Health & Employee Wellbeing Department, University of Bath, Bath, UK

**Keywords:** Researcher wellbeing, Emotionally challenging research, Secondary trauma, Vicarious trauma, Qualitative research

## Introduction

Growing evidence shows that conducting potentially emotionally challenging research may affect researchers’ wellbeing. Literature demonstrates that investigating, for example, potentially distressing topics, such as addiction (Kasseri & Tsiolis, 2025), sexual violence (Coles et al., 2014) or death and suicide (Dickson-Swift et al., 2007), or vulnerable populations, such as abused children (Perron & Hiltz, 2006) may pose challenges to researchers’ wellbeing and other aspects of life. The consequences of exposure among those interacting with traumatised individuals have variously been described as vicarious traumatisation, a concept rooted in cognitive self-development theory and centred around changes in individual’s core beliefs (Pearlman & Saakvitne, 1995) due to engagement with a traumatised person (or people) over time (British Medical Association, 2024); compassion fatigue, characterised by feelings of helplessness, confusion, isolation, numbness, avoidance, and persistent arousal (Adams et al., 2006; Cavanagh et al., 2020), in some cases with secondary trauma as a subset of compassion fatigue (Stamm, 2010); and secondary traumatic stress (STS), sometimes talked about as a syndrome of symptoms nearly identical to ‘posttraumatic stress disorder’ (‘PTSD’; Bride, 2007), that is often seen to be as a result of extreme exposure to the trauma of others (e.g., seeing the aftermath of a traumatic event or hearing it vividly told by a traumatised person). Consistent with the latter, Sprang et al. (2019) proposed that STS is characterised by four main symptom domains, aligned with the *Diagnostic and Statistical Manual*, Fifth Edition (DSM-5; American Psychiatric Association [APA],^ 2013^, ^2022^) for ‘PTSD’: recurring and distressing intrusions; avoidance of trauma-linked distressing thoughts, emotions, memories, or external cues linked to the traumatic event; a heightened state of arousal (e.g., hypervigilance, heightened startle responses, disrupted sleep); and negative alterations in cognitions and mood symptoms (e.g., change in emotion, impaired memory, withdrawal, negative cognitions about the self and others, existential threat). However, Stamm (2010, p.9) argues that whilst there are some nuanced differences between STS and vicarious trauma “there is no delineation between them sufficient to say that they are truly different”. Indeed, as Skinner et al. (in press) highlight, the literature on researcher wellbeing does not tend to state the persistence or the severity of the impacts that are discussed, so it is not always clear if what is being referred to as some kind of secondary or vicarious trauma response is in fact that (e.g., reoccurring distressing intrusions) or a short-term negative impact (e.g., distressing intrusions that are short lived). Given the lack of clarity in term usage in the literature, we instead here use the more general word *impacts*.

An increasing body of evidence documents the impact of emotionally challenging research on researchers (Batey & Szedlak, 2024; Bennett et al., 2025; Coles et al., 2014; Dickson- Swift et al., 2006, 2007; van der Merwe & Hunt, 2019; Velardo & Elliott, 2021; Williamson et al., 2020). The commonly reported emotional impacts include rage, guilt, frustration, helplessness, fear, shame, and horror (Coles et al., 2014). These emotions are often triggered by exposure to the sharing of traumatic experiences. The resultant emotional distress can lead not only to physical discomfort such as exhaustion and insomnia but also to the development of depressive and anxiety disorders (van der Merwe & Hunt, 2019; Velardo & Elliott, 2021). Researchers also commonly report feeling deeply saddened and disturbed by what they have read or heard, coupled with intense physical reactions such as crying and repeated nightmares about their participants’ stories (Coles et al., 2014; Williamson et al., 2020). Such impacts can develop at any stage of the research process, as risks are inherent in all aspects of research work, from literature review to data generation (Kiyimba & O’Reilly, 2016; Parker & O’Reilly, 2013; Velardo & Elliott, 2021; Whitt-Woosley & Sprang, 2018; Williamson et al., 2020). Nonetheless, evidence suggests that qualitative researchers may be particularly vulnerable to distress due to the methodological features of qualitative research, including close engagement with traumatised populations and prolonged exposure to distressing narratives (Kiyimba & O’Reilly, 2016; Whitt-Woosley & Sprang, 2018; Williamson et al., 2020).

While much of the literature emphasises the risks and negative consequences of emotionally challenging research, a few studies draw attention to the productive role emotions can play. Holland (2007) argues that emotions are integral to the production of knowledge, adding depth to understanding, analysis, and interpretation. Similarly, Moncur (2013) highlights that emotional engagement in sensitive contexts can foster empathy and enrich researcher–participant relationships, generating insights that might otherwise remain hidden. Further, Cole (2025) asserts that emotional reflexivity is vital, in that it aids ethical awareness, enhances analysis, deepens qualitative rigor, and renders academic careers more sustainable in the long-term. Thus, emotions can be both demanding and generative. While emotions place an added stress on researchers, they may also serve as resources that enhance the quality of research and researcher’s wellbeing.

Nevertheless, the extent to which researchers are affected varies. Several underlying factors shape researcher vulnerability in these environments. First, the literature points to challenges associated with professional boundaries between oneself and research participants (Dickson-Swift et al., 2006; Garrels et al., 2022). Several studies report that researchers may experience a moral obligation to reciprocate after participants share personal, traumatic narratives (Barbour, 2010), leading to efforts to provide comfort and support (Dickson-Swift et al., 2006; Garrels et al., 2022). However, when boundaries are maintained, researchers often feel powerless and constrained by their role (Velardo & Elliott, 2021). Another contributing factor to vulnerability to distress is the researcher’s background, experiences, and personal trauma in relation to their research topic. van der Merwe and Hunt (2019) found that researchers with similar backgrounds to the traumatised population are more likely to experience ‘overidentification’ and exhaustion due to shared primary trauma, leading to heightened feelings of helplessness, anger and grief. Lastly, questions have been raised about whether the impact of emotional work diminishes with age and experience. Recent studies have challenged this assumption. For example, Williamson et al. (2020) indicated that the impact of emotional work may lead to cumulative trauma. Starcher and Stolzenberg (2020) found that the intensity of STS is largely unrelated to a researcher’s age or experience. Instead, they suggest that factors such as organisational support and job satisfaction may play a more significant role in the impact of emotional work.

### Present Study

Extensive research documents the experiences of counsellors, social workers, healthcare professionals, first responders, and humanitarian workers impacted by emotionally challenging work (e.g., Coles et al., 2014; Molnar et al., 2020; Ogińska-Bulik et al., 2021; Sprang et al., 2011; Strohmeier & Scholte, 2015). These studies shed light on factors influencing individual responses, supporting the development of various prevention and intervention methods for individuals in various helping professions. While there is a growing body of evidence on researchers’ wellbeing, it predominantly compromises studies often confined to specific disciplines or focused on particular topics, such as sexual violence (Coles et al., 2014; Kennedy et al., 2014). Moreover, existing research largely focuses on the negative impacts of emotionally challenging research, with limited attention to the potential of positive impacts (e.g., Skinner et al., 2023). Building on existing evidence that emotions can also enrich understanding, empathy, and analytic depth, the present study recognises emotions as both challenging and potentially helpful in the research process. We therefore aimed to fill this gap by investigating the experiences of academic researchers undertaking emotionally challenging research across a range of topics and disciplines. Specifically, the study aims to explore both the positive and negative impacts of such work and to identify factors that may increase researchers’ vulnerability to its adverse effects.

## Methods

### Participants

The study included 31 university staff members, 19 females and 11 males (one participant did not complete the pre-interview questionnaire). Participants represented six different departments and, on average, had been involved in sensitive research for 12 years, with experience ranging from as little as three months to as much as 35 years. Whilst the sample was diverse in terms of nationality/country of origin, most of the participants were white; five considered themselves disabled; one indicated they were LGBTQi. To protect participant confidentiality, we can only provide an overview of the broad research areas they focus on, which include violence, abuse, conflict, mental and physical health, marginalized and vulnerable populations, care, discrimination, and social/criminal injustice. Therefore, many participants worked across multiple sensitive topics. See Table 1 for more participant demographic information.

**Table 1:** Demographic characteristics of the sample

Variable	*N* = 30
Age	25–60 (M = 42; *SD* = 9.8)
Gender	
Female	19 (63%)
Male	11 (37%)
Job title	
Lecturer	8
Professor	3
Reader	3
Research Assistant	2
Research Associate	6
Research Fellow	1
Senior Lecturer	7
Dis-ability	
No	25
Yes	5

One participant did not complete the pre-interview questionnaire

### Sampling and Procedure

Thirty-one semi-structured interviews were conducted with researchers engaged in or who had previously undertaken research on potentially emotionally challenging topics. The study employed a purposeful and multi-stage sampling method to select participants. The target population consisted of staff members employed by the Faculty of Humanities and Social Sciences at the University of Bath on a research contract or research and teaching contract and who were currently undertaking or had previously undertaken research on potentially distressing topics. The University is English, research intensive, ranked 132nd in the QS World University Rankings (QS TopUniversities, 2025), a top 10 UK institution (e.g., ranked 7th best university of the year in The Times and Sunday Times Good University Guide; The Times and Sunday Times, n.d.) and is likely to have a lower teaching load than post-1992 universities in England. Potential participants were identified based on their engagement in or completion of research on potentially distressing topics, considering their research topic of interest, and the nature of the research. All students were excluded from the study except lecturers/researchers currently undertaking a part-time PhD. Researchers were initially identified via information on the University of Bath's website between April 19th and April 27th, 2023, and were contacted via email and invited to participate in the study. The invitation email included a Participant Information Sheet outlining the purpose of the study, what participation would involve, and ethical considerations. Researchers who wished to participate expressed their interest by replying to the email. Initially, 38 individuals were invited to participate in the study, of which 15 participated. Additionally, 29 more researchers who met the eligibility criteria were identified through snowball sampling and invited via email, of whom 14 participated. Furthermore, a faculty-wide email was sent on June 22^nd,^ from which two more participants were recruited. The latter two sampling methods ensured that more junior/temporary staff who did not have a webpage or that we had not identified as undertaking emotionally challenging research could choose to participate. Prior to the interviews, all participants received an information sheet, were asked to sign a consent form and complete a pre-interview questionnaire on the survey platform QuestionPro. Participation in the interviews was completely voluntary. Participants were informed that they could pause or stop the interview at any time without needing to provide any reason, were informally debriefed^1^ (see Appendix A) at the end of the interview (e.g., ended on their positive coping strategies, provided a verbal summary of what they had said and asked if this was accurate, informed them of support services they might access, and provided with written information on how to contact them), and could withdraw their data for up to two weeks following the interview. The research team also implemented strategies to manage researcher wellbeing and maintain analytical rigour, including developing a wellbeing plan, undertaking training on how to respond if a participant became distressed, holding regular informal debriefing sessions following interviews, and engaging in monthly independent wellbeing supervision (also called ‘clinical supervision’ see Skinner et al., 2025 for definition). All research participants were also offered, in their debriefing and in a follow up email, 10–20 sessions of specialist trauma focused counselling free through the project if they needed it. This was taken up by three participants. Having this resource available also eased some of the concern the team felt about the mental health of some of the participants, thus aiding our own wellbeing. The interviews were guided by a previously developed topic guide (see Appendix A), with 30 conducted via Teams and one face-to-face. Interviews were conducted from May to July 2023. On average, interviews lasted 54 min, were audio-recorded, and professionally transcribed. Ethics approval was received from the University of Bath Ethics Committee (Ref: S23012) .

### Data analysis and Trustworthiness

Data was analysed using thematic analysis, primarily following the approach outlined by Braun and Clarke (2006) and including investigator triangulation to aid trustworthiness (see Korstjens & Moser’s, 2018 development of Lincoln & Guba, 1985). Brance and Skinner first familiarised themselves with the data by listening to the audio recordings, reading the transcripts in detail (Brance only), and making preliminary notes on initial observations and patterns across interviews. They then generated initial codes independently while closely examining five of the transcripts. Codes were then compared, discussed, refined, collated and organised into broader categories, which formed the basis for the development of prospective themes and the initial codebook. Brance then applied these codes to the remaining transcripts in NVivo 14. Any new codes developed in subsequent coding, or uncertainty about coding of a section of transcript, were also discussed by Brance and Skinner and added to the codebook. They shared the initial themes, illustrative extracts and initial analysis with the wider research team, whose feedback informed further revisions and helped refine the analysis to more closely address the research question. Through this iterative process, the team reviewed and refined the themes collaboratively ensuring that the final themes and representative extracts reflected a shared interpretative understanding of the data. The researchers then defined and named the themes to ensure that each captured patterned meaning across the dataset and contributed to a coherent analytic narrative. They then selected key extracts to illustrate the central arguments of each theme.

The credibility of the analysis was further enhanced through prolonged engagement (Korstjens & Moser, 2018), in that the authors work in the same institution as the participants and are highly familiar with the context under investigation; and *member checks* were made through several avenues, including participants being able to give feedback on the project findings and report before general release (via direct emails and face-to-face discussions), and presentations within the University and externally. Whilst the initial member checks produced limited feedback other than a positive response, our ongoing engagement with the wider academic community has helped to further refine our analysis. For example, our initial report of the data in verbal presentations and an initial draft of this paper was highlighted by some audience members and an independent reviewer as overly focused on negative impacts. Whilst we did have a positive impacts theme, we had not included this data in this earlier work. This feedback, alongside returning to the data, helped us correct this vital omission.

## Results

**Fig. 1:**
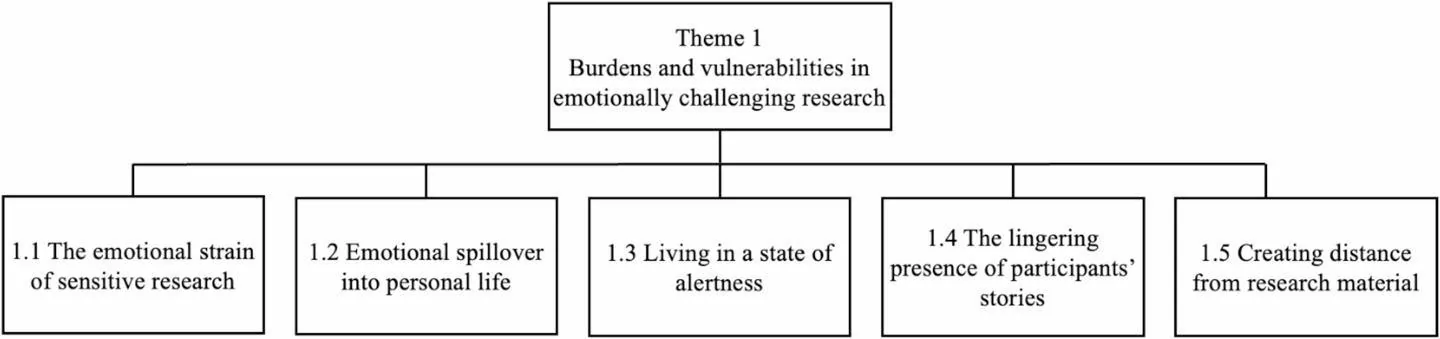
Overview of Subthemes within Theme 1

**Fig. 2:**
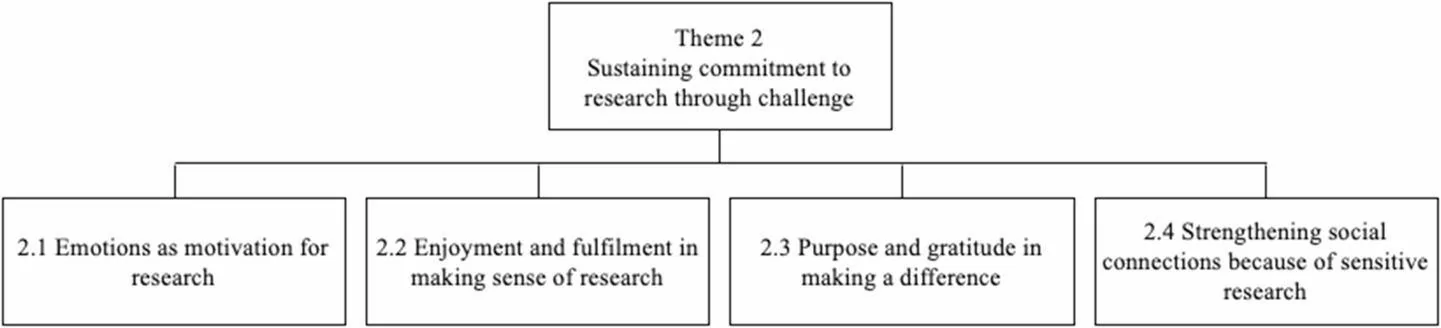
Overview of Subthemes within Theme 2

The impacts findings consisted of two dominant themes that we have divided into subthemes. Theme 1 focuses on burdens and vulnerabilities in emotionally challenging research and is illustrated in Fig. 1. The subthemes therein are: the emotional strain of sensitive research; emotional spillover into personal life; living in a state of alertness; the lingering presence of participants’ stories; and creating distance from research material. The second theme, illustrated in Fig. 2, is more positive and related to sustaining commitment to research through challenge, including the subthemes of: emotions as motivation for research; enjoyment and fulfilment in making sense of research; purpose and gratitude in attempting to make a difference; and strengthening social connections because of sensitive research.

### Theme 1: Burdens and Vulnerabilities in Emotionally Challenging Research

Engaging in emotionally challenging research often had notable implications for many participants, who described a range of negative impacts on their wellbeing. Across different topics and disciplines, participants reported experiences consistent with symptoms often associated with secondary or vicarious trauma, and in a few cases, researchers experienced direct trauma when they themselves became targets of abuse or violence in the course of their work. However, participants’ accounts also revealed considerable variation in how these impacts were experienced and interpreted. Some participants reported being rarely or minimally affected, whereas others described more substantial negative influences on their psychological wellbeing or their capacity to function. Even within individuals, experiences varied depending on workload, the specific nature of the research data, or what was happening in their personal lives at the time.

Participants’ accounts highlighted several factors shaping their vulnerability to the negative impacts of emotionally challenging research. Firstly, limited control over research topics and study methods often increased the emotional burden of the work, particularly among early-career and contract researchers who had little choice over the nature of their work. Secondly, prolonged engagement in such research, particularly in the context of extended or fixed-terms research contracts, contributed to increased vulnerability over time. Thirdly, limited training and knowledge about coping with the adverse effects of emotionally challenging research was identified as contributing to vulnerability, especially among those with limited prior research experience or access to appropriate support. Fourthly, shared experience with participants could intensify emotional engagement with research material, sometimes triggering personal trauma and heightening emotional responses. For example, Participant 16 described what shared experience feels like: *“It’s particularly … like somebody’s touching or putting salt in a wound that we have.”* Finally, personal life challenges unrelated to the research topic could further reduce researchers’ capacity to engage with emotionally demanding work. Participants shared how various life events, such as becoming a parent, facing personal or work relationship issues, as well as mental and/or physical health concerns/dis-ability, strained their capacity to engage with specific research topics or aspects of work. While these factors were linked to increased vulnerability, their absence did not necessarily imply the absence of negative impacts. Rather, participants’ accounts suggest that individual, professional, and contextual factors interact in shaping responses to emotionally challenging research. Although some participants who reported fewer negative impacts described having greater control over their work, more research experience, or prior training (often from outside the university), these patterns were not consistent across all accounts and were not systematically examined in the current study. The following subthemes elaborate on how the participants experienced and expressed the negative impacts of their research work.

### Subtheme 1.1: The Emotional Strain of Sensitive Research

Participants described a range of emotions they experienced as a result of their work, including distress, anxiety, feeling drained, disgust, guilt, frustration, powerlessness, sadness, and fear. For some, feelings of sadness went beyond being an immediate response and extended into more profound experiences of feeling depressed at times. For example, Participant 21 recalled: “*I remember sitting on a bench*,* and I couldn’t enjoy anything*,* […] I was totally out of it in many ways*,* and I couldn’t even enjoy eating an ice cream!”*

Guilt emerged as a complex emotional response. Participants mentioned experiencing guilt for various reasons, including feeling conflicted about taking time for self-care after distressing interviews, feeling positive emotions, or engaging in usual activities following distressing research encounters. Some participants also felt guilt over their inability to assist participants beyond their role as researchers due to professional boundaries; and some felt a sense of responsibility for the wellbeing and safety of those they interviewed.

*One thing that I often think about is*,* as a result of that*,* have I put that particular* [participant] *at more risk. […] So it goes back to those ethical questions. […]. I don’t know*,* does that* [participant] *become even more a target of discrimination because I went there? And I don’t know that because I don’t do the follow-up.* (Participant 24)

Participants also shared that a significant challenge in the researcher’s professional role is handling emotional reactions to the potentially traumatising stories shared by their interviewees. While some participants felt that displaying emotions, such as crying, is inappropriate and should be avoided, others viewed emotional reactions as a way to demonstrate empathy. Two participants specifically recounted instances where breaking down was considered inappropriate, later feeling profound guilt and concern about the additional burden they may have placed on their participants. Participant 11 shared:

*I don’t think that was appropriate at all. Because it was proper crying*,* you know*,* it wasn’t just showing emotion*,* it was actually* [mimics sobbing] *onto other people who had given up their time to talk about their* [experience]. *They were handling their own issues. They didn’t need to then be handling mine as well.*

This underlines a critical aspect of the researcher’s role, which is to balance empathy with the primary responsibility of attentively listening to participants without burdening them with their personal issues. Feelings of guilt also extended to the perceived lack of policy and practice impact of researchers’ work. Participants expressed their sense of helplessness and powerlessness regarding the influence they could exert, often feeling that their research made only a small contribution in comparison to the substantial societal, political, or economic changes needed.

For some, the emotional strain of engaging with distressing research material was not contained within the research process but extended into how they perceived their work, their participants, and the wider world. For example, Participant 15 recounted a significant shift in their perception:

*The most acute part is when you’re […] seeing the things that you write about*,* and you read about*,* […] there’s a real helplessness in those moments*,* and a real sort of despair […] feeling what I was doing is very meaningless and such a drop in what needs to be done […]*.

Similarly, Participant 16 reflected: *“It was moving*,* and it was upsetting. And it was kind of difficult to see what kind of suffering exists in the world that we live in. And it’s never very far away from us.”*

Therefore, one participant spoke proactively about wanting to leave not only emotionally challenging work but academia and research entirely:

*When you’re doing a research job where*,* you know*,* it kind of leaves you having dark thoughts in the middle of the night*,* you do have to think: Well*,* is this really worth it*,* you know? So I’ve been doing a whole load of questioning. I would say*,* since XXX project. […] It all feels very dry and burdensome. So I’ve been*,* for the last two years*,* very proactive about applying for jobs outside of academia. I’m writing a job application right now*,* actually. Because I just think*,* if this is the future*,* it’s not worth it. […] I can start having fun!* (Participant 22)

For some, the emotional toll was not only psychological but also physically felt. Fear, despair, and anxiety were described as embodied experiences that manifest in sensations such as fatigue, chest tightness, nausea, or changes in voice when talking about the research. For instance, reflecting on their experience while collecting data, Participant 15 elaborated: *“A sort of fear*,* I guess a physical repulsion […] that this could be possible today*,* that people would do this. […] I would feel it in my body […] remember feeling sort of quite sick.”*

### Subtheme 1.2: Emotional Spillover into Personal life

Some participants described how shifts in their emotional states, including increased irritation, stress or anxiety, were affecting their family relationships, impacting interactions with partners and children. Others worried that discussing their research might place an emotional burden on family, friends or colleagues, leading them to limit what they shared in order to protect others from distress. For example:

*I realised I was coming home and I … and it was just kind of whirring round my head. And I couldn’t tell anyone about what was in my head*,* but I needed a way to kind of decompress because when […my child is…] at home*,* [… they are…] not aware of what’s going through my head […they want…]to play or […] to talk or watch TV or play games or you know we’re doing [their] homework. So I knew I needed something to just give me space … to decompress.* (Participant 25)

Participant 1’s partner temporarily moved out of their home to avoid exposure to the project data. However, they ensured Participant 1’s wellbeing before doing so, highlighting the supportive role that partners often play in emotionally challenging work:

[during transcription] *my husband had to move out*,* […] that was an extended period because*,* as you can imagine*,* that took a long time to do and have great sympathy for him in not hearing that in the house. And but*,* obviously*,* he didn’t leave before really*,* you know*,* checking that I had a self-care plan.* (Participant 1)

Alongside outward effects, some participants also described more inward responses that affected how they related to others. Three participants described noticing becoming “colder” towards issues raised by family, friends or students, with everyday concerns seeming less significant in comparison to the trauma they encountered through research. For example:

*It probably makes me cold.* […] *I think a danger of this kind of research is when someone has a problem which to them is quite important*,* you know a student*, [such problems] *don’t seem as significant* […] *but I try and act like they are!* (Participant 28)

While ‘coldness’ was described as a lived impact on relationships, participants also reflected on ‘numbness’ more broadly as a warning sign. Many regarded it as an indicator that one should consider stepping back from their research upon reaching this state. However, two participants suggested that ‘numbness’ or ‘coldness’ could also serve as a coping mechanism, allowing for a degree of emotional distance that helped them manage the demands of their work. This highlights how some separation from the experiences portrayed in the data was seen as both a risk and, for a few, a form of self-protection.

### Subtheme 1.3: Living in a State of Alertness

For some participants, engaging with emotionally challenging research led to sleep disturbances or difficulty concentrating, often linked to qualitative aspects of their work, such as reading interview transcripts. For others, these disruptions were triggered by revisiting distressing aspects of the research or by concerns for the wellbeing of participants and research team members, reflecting a sense of responsibility that extended beyond their own experiences. Even one participant who claimed to be generally ‘unaffected’ by their research still described experiencing some disturbances in their sleep. For example, *The only sleepless nights I’ve had about my research have been where other people have been interviewing* [participants]. *And I’ve worried about: ‘will my researchers be OK?’*,* ‘could something happen to a* [participant]*?’* (Participant 25).

Beyond personal reactivity, a few participants described instances of self-destructive behavior, for example, one participant linked their anger to episodes of excessive drinking. Furthermore, while some participants acknowledged general irritability and anger, nearly all interviewees felt angry about the trauma and suffering endured by their participants and the lack of positive change in service/ state policies and practices. For example,

*One of the issues is actually managing anger. And*,* yeah*,* resentment*,* you know*,* seeing what goes on and the lack of accountability that can be very frustrating*,* upsetting*,* infuriating. Yeah. One of the things I’ve had to manage over the years is how one deals with that rage that*,* you know*,* that sense of injustice.* (Participant 8)

### Subtheme 1.4: The Lingering Presence of Participants’ Stories

Many participants recalled thoughts and memories that lingered vividly in their minds, sometimes resurfacing even years after exposure to others’ trauma. Participant 22 described such experiences as mentally *“polluting”.* For some, these intrusive thoughts were also linked to their own experiences of primary trauma:

*Listening to other people’s suffering reminds me of my own*,* and some stuff that*,* you know*,* I’ve kept buried for a long time. […] it just sort of lands on you*,* doesn’t it? It comes up behind your back*,* and the thoughts come into your head! (*Participant 22)

A few participants also described experiencing nightmares, which typically arose shortly after engaging in distressing material: *“I was getting like nightmares. I was having problems with sleeping. Nothing super serious*,* but I noticed that I was being disturbed by the data that I was working on.”* (Participant 4). Notably, several participants reported an increase in intrusive thoughts during the COVID-19 pandemic. The imposed restrictions on day-to-day social activities, which had previously helped participants distance themselves from the distressing aspects of their work, left them with more time to dwell on it.

*The lockdown time* […] *really gave you too much time to ruminate on*,* to suddenly be thrust into kind of trauma and* [topic]. […] *normally*,* I would just do other things*, […] *go out with friends.* […] *do all the kind of social things* […]. (Participant 17)

### Subtheme 1.5: Creating Distance from Research Material

For some participants, the emotional strain of their research led them to step back from the material either temporarily or deliberately. Rather than reflecting a structured coping strategy, these behaviours often resembled withdrawal or avoidance responses linked to the emotional demands of the work. Participants described moments of disengagement from data or fieldwork as a way of managing the intensity of exposure to distressing material. Participant 22 explained:

*Yeah*,* it’s called avoidance* [of data analysis] *by getting up and just going*, oh f*ck this,* I’m off to* [do a hobby]*! But then equally*,* doing that classic thing that you’re not really enjoying your time* [doing a hobby] *because you feel guilty that you’re not at your desk coding your data!*

Whilst Participant 22 described avoidance accompanied by feelings of guilt, others spoke about stepping back from emotionally challenging material in different ways. For example, some participants described taking breaks after distressing interviews or creating temporal separation between time spent engaging with research material and the rest of their daily life. Such distancing was particularly highlighted by those involved in fieldwork, who described needing to step away from the emotional intensity of the work once they returned from the field. As Participant 28 explained:

*I mean sometimes just like you know*,* going for walks*,* or trying to like leave* […] *like you know*,* having a time where this is where I’m looking at* [research] *material and then it ends*,* and then*,* you know*,* I go for a walk or something* […] *that’s like a buffer between the next like part of my life.*

### Theme 2: Sustaining Commitment to Research Through Challenge

Although existing literature largely focuses on the emotional burdens of undertaking emotionally challenging research, participants in the current study also articulated a range of positive impacts. Many described how some of the same emotions that made their work challenging also motivated them. They also enjoyed their work and found the process of sense-making fulfilling; it gave them a sense of purpose, fostered gratitude for their own lives, and deepened their connection to the topics they research. As indicated in Fig. 2, these four subthemes all helped them to sustain their commitment to their work. Across interviews, participants described their experiences in terms of love, enjoyment, passion, and reward, highlighting that even within contexts marked by sadness, anger, or frustration, research can remain deeply fulfilling. The following subthemes elaborate on how the positive impacts were experienced and expressed in participants’ accounts.

### Subtheme 2.1: Emotions as Motivation for Research

Participants highlighted how emotions often framed as ‘negative’ could in fact support their commitment to research. Feelings of sadness, fear, and anger were often described as appropriate, meaning-making responses. For some, sadness enabled deeper understanding of participants’ lives. As Participant 1 explained: “*It sort of moves you for a while*,* and you stay moved for a while. I don’t necessarily though personally see that as a bad thing. I sometimes actually feel it as a…maybe a good thing*.”

Feelings of fear and anxiety were frequently normalised, particularly in the context of fieldwork when uncertainty about another researcher’s safety was present. For example, when colleagues were temporarily unreachable, Participant 28 recalled:

*In those moments they can’t communicate with you.* […] *they’re kind of emotional times* […] *I think it’s like appropriate*,* right*,* if you have*,* if you’re waiting*,* it doesn’t feel like a bad emotion*,* it feels like an appropriate fear or worry or anxiety that then passes*.

Anger was the most frequently discussed emotion and generally seen as positive and often tied to why participants pursued their research. As Participant 23 noted: “*I don’t know whether it was distressing*,* it did make me quite a bit angry I think*,* but also it highlighted how important it is for us to be doing this research* […].*”* For others, anger was central to their identity as a researcher:

*I would like to think again*,* you know*,* as whenever I finished doing research that people will have looked at me as doing research on topics that I was angry about. And therefore wanted to change. And that’s what I do*,* I’ve got particular ways of doing research because that’s what drives me doing research. And it’s probably the reason I’m doing research.* (Participant 14)

Similarly, Participant 15 said: *“I feel like I’m fueled by the anger that I get from knowing that this is the state of things and yeah*,* I think that’s just how I’m wired in that. That’s a motivating thing for me wanting to change that.”* However, participants also reflected on the risks of unmanaged anger. For example, Participant 2 described anger as “*absolutely necessary*,” yet warned that without care it could become “*despair*” or “*cynicism*”.

### Subtheme 2.2: Enjoyment and Fulfilment in Making Sense of Research

Several participants spoke explicitly of “loving” their research or finding it “enjoyable” despite, or sometimes because of, its emotional intensity. For some, these feelings were longstanding and reflected their continuing interest in the research area. Others linked a sense of passion for research to their own history of trauma or other personal experiences. For Participant 26, this passion was described in visceral terms: “*I mean I love it. I am learning so much*,* and it’s like it’s giving a lot*,* yeah*,* to my life. Like the topic itself is like super fascinating…this is also where my heart* [is].*”* Participant 29 similarly reflected: *“I mean I love what I do*,* it’s cool*,* yeah. I mean how have I found working in this … great*,* I love it*,* it’s cool.”*

Enjoyment was not only associated with the content of the research but also with research processes. For example, Participant 31 explained that they “*really enjoy writing and analysing*” because analysis was *“like a jigsaw puzzle…of making sense”* while writing was experienced as *“very cathartic and very peaceful.”* For this participant, making sense of complex qualitative material was in itself a positive, and even therapeutic, experience.

Similarly, participants often described the intellectual stimulation that emotionally challenging research afforded. Participants frequently used terms such as “interesting,” “fascinating,” and “engaging” to describe their encounters with data and research participants. Participant 3 described: *“I just enjoy talking to people and learning from people and*,* you know*,* having a sense then I can kind of contribute maybe a bit to making their stories heard in a small way.”* Similarly, Participant 12 reflected: *“I find this enjoyable… it’s what interests me.”* For others, satisfaction came from engaging in complex social or political problems through their research. Participant 14 highlighted this explicitly: *“There are so many problems that need solving both*,* I mean*,* politically*,* but scientifically as well*,* especially about* [research topic]. *There’s just so much we don’t know and that is very motivating.”* Participant 18 similarly emphasised the rewards of conceptual advances: *“Those moments when you realize that*,* ‘hey*,* now I understand something deeper.’ Those are fantastic moments.”*

### Subtheme 2.3: Purpose and Gratitude in Making a Difference

The positive impacts of participants’ research were often tied to the sense of purpose and meaning it provided. Doing work that “mattered” was repeatedly emphasised, even where participants acknowledged the limitations of the research impact. Participant 16 described: “*I feel that I found them very rewarding*,* and they feel very relevant* […] *They have definitely been sometimes quite challenging to research for sure. They’ve definitely been emotive and sometimes distressing. But…it feels like it matters.”* Similarly, Participant 2 noted:

*Mostly rewarding overall*,* I feel like I’m doing something*,* but hopefully it matters a little bit.* […] *I’m not blind to the fact that my impact on on all of these is very minimal*,* but I still feel that I’m doing something that matters. That is the right thing to do.*

The sense of making a difference extended beyond personal fulfilment and was often explicitly tied to social contribution. For Participant 6, the research was meaningful because it offered a way to capture and share the experiences of marginalised communities:

*I don’t for one minute suggest that my research has an impact on those people’s lives or changes them for the better. But I suppose what I can do is kind of capture the stories and the narratives and the experience of these types of people to kind of help and think about how we can support them and perhaps address some of the issues that they face.*

Alongside a sense of purpose, many participants expressed gratitude for the opportunity to conduct emotionally challenging research. Several described feeling privileged to be entrusted with personal and emotionally painful stories. Participant 31 reflected: *“I feel very fortunate* […] *I’ve felt very free and very able to just pursue the things that I think are worth researching.”* Participant 13 similarly said:

*I think I feel very privileged to be in a position where people*,* who’ve never met me* […] *feel that they can pretty much open up and tell me really personal stuff*,* often linked to some of the most awful things that have happened to them.*

For others, gratitude was tied to recognising their own positionality and privilege in contrast to participants’ hardships. As Participant 15 noted: *“Sometimes it makes me feel incredibly lucky that this is not my life and not having to go through these things.”* Similarly, Participant 28 said: *“It also just kind of makes you grateful for everything you’ve got in the moment.”*

### Subtheme 2.4: Strengthening Social Connections Because of Sensitive Research

For some participants, the positive impacts of conducting emotionally challenging research extended beyond their individual sense of purpose and wellbeing, influencing how they related to others in their personal and professional lives. For example, research experiences were described as shaping parenting practices, particularly in terms of raising awareness about inequality and human differences. Participant 23 reflected on how their work informed conversations with their children about racism and sexism:

*I remember having sort of discussions that you know people might be different. Because I realised that unless I tell my children that someone might not be white*,* then it might not cross their mind.* […] *So in some ways I thought this probably affected a little bit how I talk to them about this. I always talk to them about things*,* kind of other dimensions that people differ*,* because another thing close to my heart but it’s not as controversial*,* is gender. And gender inequalities in general.*

Beyond parenting, a few participants reported that their research experiences shaped the way they thought about relationships in the workplace. For example, Participant 22 described how listening to participants’ accounts of suffering highlighted the importance of connection with others:

*I listen to people who are* [suffering from the topic of the research], *and they tell me* […] *it’s about your quality of relationships. Well where’s the quality of relationships if all your working relationships are just remote? So it does make me think*,* actually yeah*,* I’d rather build friendships and kind of relationships in my working life that’s more face to face.*

## Discussion

A growing body of research highlights the numerous challenges researchers face when investigating emotionally challenging topics, indicating that the distressing nature of such research can lead to a range of psychological and physiological symptoms (e.g., Batey & Szedlak, 2024; Coles et al., 2014; Starcher & Stolzenberg, 2020; Williamson et al., 2020). While a comprehensive review by Cieslak et al. (2014) demonstrated that secondary trauma is widely recognised as a significant, common, and potentially impairing response among those working with clients or recipients exposed to traumatic stressors, the specific challenges and impacts faced by researchers across a range of academic disciplines and topics remain unclear. Although a few studies recognize that engaging in emotionally demanding research can also have positive or transformative effects on researchers, evidence remains limited. Addressing these research gaps, the current study sought to examine both the negative and the positive impacts of conducting emotionally challenging research within a Faculty of Humanities and Social Sciences at one university.

Findings from the study highlight the complexity of the impacts potentially distressing research can have on researchers. Participants frequently described symptoms associated with secondary and vicarious trauma, highlighting the toll research work can have on individual wellbeing. These findings resonate strongly with theoretical descriptions of secondary traumatic stress and compassion fatigue outlined in the literature (Adams et al., 2006; Cavanagh et al., 2020; Sprang et al., 2019), particularly in relation to recurring emotional distress, withdrawal, and heightened emotional responses following exposure to traumatic narratives. Existing literature indicates that researcher’s personal background and experiences are risk factors for increased detrimental effects and potential for re-traumatisation (van der Merwe & Hunt, 2019). Building upon this, the current study identified additional key factors that contribute to researcher vulnerability. Particularly prevalent among early-career and long-term contract researchers, the lack of control over research choices is associated with various symptoms, including feeling emotionally and physically drained. The lack of control, in addition to the finding that inadequate training in coping strategies leaves researchers, especially those at early career stages or on precarious contracts, more susceptible to trauma, suggests a critical need for comprehensive training programmes, improved supervision/management and measures to address job insecurity. Additionally, personal life events, such as parenting, could further exacerbate researchers’ vulnerability. This finding extends the discussion about researcher wellbeing beyond work-related challenges to also consider the influence of personal life circumstances.

The range of emotional impacts reported by participants are in line with previous literature demonstrating that sensitive research work can put researchers at risk of developing ‘PTSD’-like symptoms. Whilst we are not able, from the data presented here, to indicate the severity or longevity of the impacts on participants, many are consistent with the symptom domains of secondary traumatic stress described by Sprang et al. (2019), including intrusions, emotional exhaustion, and avoidance responses associated with prolonged engagement with distressing material. The findings build upon and reinforce the existing body of research on the psychological impact of trauma work among helping professionals (Ogińska-Bulik et al., 2021), extending its relevance to include those engaged in conducting potentially traumatising research. Notably, while emotional connectedness and responsibility are inherent in both researcher and professional roles (e.g., psychotherapists or social workers), researchers’ capacity to provide support and guidance to their participants is limited, potentially intensifying negative impacts on researchers. Existing literature highlights that researchers often feel constrained by their role due to professional boundaries, resulting in feelings of powerlessness and helplessness (Velardo & Elliott, 2021). Our study indicates that researchers frequently experienced a profound sense of guilt for being unable to offer guidance to participants beyond their role as a researcher. This tension between emotional engagement and professional boundaries has been widely noted in research on sensitive qualitative work, where researchers must navigate strong emotional responses while maintaining appropriate professional roles (Dickson-Swift et al., 2006). Moreover, they reported feelings of anger and frustration for their inability to effect immediate changes for participants after witnessing their suffering. Furthermore, the extension of the impact on social relationships and career choices is particularly noteworthy. The strain on personal relationships and the decision of some researchers to shift careers to preserve their wellbeing highlight the long-term consequences of exposure to potentially traumatic content.

While much of the existing literature points to the burdens of emotionally challenging research (e.g., Batey & Szedlak, 2024; Bennett et al., 2025), current findings demonstrate that emotions can also be helpful in shaping how researchers engage in their work and contributing to a sense of meaning and purpose. Emotions often labelled as ‘negative’, such as sadness, fear, or anger, were frequently described as appropriate and meaning-making responses to participants’ experiences. This aligns with arguments that emotional engagement in qualitative research can deepen analytic insight and ethical responsiveness rather than undermine rigour (Holland, 2007; Moncur, 2013). Recent work similarly emphasises that emotional reflexivity can strengthen relational understanding, ethical sensitivity, and analytic depth in qualitative research (Cole, 2025). Rather than undermining research rigour, these emotions fostered sensitivity, informed judgement, and ability to ask difficult research questions and motivated researchers to confront the injustices revealed through their work.

Participants also talked about passion for their research, describing the privilege of being entrusted with personal and often emotionally painful accounts, and the satisfaction of making sense of complex material. For many, an important source of motivation and commitment to their work was the idea that their research mattered, even where immediate impact was limited. This is consistent with literature that recongises emotion as an integral part of research and draws attention to the emotional work involved in engaging with distressing research material (Holland, 2007). In this sense, the findings reinforce theoretical perspectives that conceptualise emotion as both a potential source of vulnerability and a resource for knowledge production. Cole (2025) similarly argues that emotional engagement should be understood not as a methodological liability but as a potential asset that can enrich qualitative inquiry when accompanied by reflexive practice. Therefore, it can be argued that emotions function as resources for knowledge production, rather than solely a risk to wellbeing, provided it is acknowledged and supported through supervision, informal debriefing, peer support, and clear institutional guidelines (Skinner et al., 2025). However, recognising the positive impacts does not diminish the seriousness of risk among those engaging in this work. It rather highlights that the same emotional processes that can threaten wellbeing also have the potential to support more compassionate, ethically responsible, and analytically rich research practice when embedded in a supportive research environment.

This research has several key implications for research practice and policy. Primarily, it highlights the need for a collaborative approach in addressing researcher wellbeing, involving not only the researchers but also their supervisors, managers, and the institutions they are part of. To mitigate the risks associated with potentially traumatising research, it is essential to move beyond seeing the wellbeing of researchers as solely their individual responsibility. While individual self-care is important, it alone is insufficient in the face of potentially traumatising research. A supportive working environment and community are crucial. If the moral imperative to create such an environment alone is not enough, in England, it is a legal requirement under the Health and Safety at Work Act 1974, Management of Health and Safety at Work Regulations 1999 to risk assess and safety plan for psychological risks associated with employment (similar legislation exist across the Europe Union). It also makes good business sense to look after employees, particularly when a substantial investment of time and money has been made to get them to the stage of being able to do emotionally demanding research.

Practical steps include training in coping strategies and the provision of accessible mental health resources for researchers working on sensitive topics. The development of guidelines and ethical considerations around the conduct of sensitive research is also imperative. Such guidelines could include protocols for debriefing, peer support groups and networks, and regular wellbeing check-ins, ensuring that researchers are fully aware of the challenges they may encounter and are equipped with strategies to address them. Formalising these practices, which are already established in other caring professions, would create opportunities for informal debriefing and allow researchers to process their emotions without breaching professional boundaries or over-relying on informal support from family and friends. Ethics processes could further embed this shift by requiring that potential risks are explicitly identified, and that clear mitigation plans are in place.

Emerging work in this area has begun to outline what such institutional structures may look like in practice. For example, Skinner et al. (2025) propose a staged “Bronze, Silver, Gold” approach for supporting researchers undertaking emotionally challenging work. At a minimum level, institutions should provide awareness raising activities, written guidance and clear referral pathways for researchers who may experience distress (SVRI, 2015). More developed responses may include specialised training, funded Researcher Wellbeing Plans, opportunities for informal debriefing, and access to independent wellbeing supervision. A range of practical resources to support these approaches is beginning to emerge across the sector, including researcher wellbeing plans (Zschomler et al., 2023), guidance for responding to participant or researcher distress, and risk assessment tools designed specifically for emotionally challenging research (see link to Researcher Wellbeing Project resources at the end of this article). Drawing on and adapting such existing resources may help institutions move from general recognition of the issue toward the implementation of concrete and sustainable support structures.

More broadly, as indicated by Skinner et al. (2025), creating a research culture that openly acknowledges and addresses challenges inherent in research work is vital. This involves raising awareness among employers and funders of the impact on researchers and advocating for necessary measures to support their wellbeing. Such efforts may include integrating researcher wellbeing considerations into ethics review and project planning processes, establishing peer-support networks, and ensuring that supervisors and research leaders are trained to recognise and respond to the potential impacts of emotionally demanding research (e.g., Garrels et al., 2022; Silverio et al., 2022; Whitt-Woosley & Sprang, 2018). Overall, the findings highlight the need to re-evaluate research practices and policies, moving beyond individual responsibility towards shared institutional obligation. By addressing these challenges, universities can foster a more sustainable research environment, ensuring that sensitive research does not come at the expense of those who dedicate themselves to this work.

The study is not without limitations. Firstly, while substantial for a qualitative study, the sample size of 31 participants interviewed does not fully encompass the diversity of experiences and perspectives among the broader population of researchers engaged in potentially distressing research topics. Additionally, the focus on researchers from a single university may introduce sampling bias, as the experiences and support systems available at this institution may differ from those at other universities or research organisations. However, it is worth noting that appropriate frameworks to support affected individuals are often lacking, both institutionally, nationally in the UK and internationally, further underscoring the significance of exploring the experiences and wellbeing of researchers in potentially traumatising research fields. Furthermore, while the analysis identified factors described in relation to vulnerability to negative impacts, it did not systematically examine protective factors or the conditions under which such impacts may be mitigated. Although coping strategies and sources of support are explored in a related paper (Skinner et al., 2025), the present study did not aim to determine how or when such factors prevent the emergence of negative impacts. Future research could therefore draw on longitudinal designs to better capture the dynamics of burdens and benefits as they unfold in researchers’ careers over time.

## Conclusion

Most participants were passionate about their work, yet researching emotionally challenging topics had numerous negative impacts. These impacts included emotional symptoms and existential crises linked to the systemic injustices of human suffering, alongside disruptions in the researchers’ social relationships and careers. Even some of those who claimed no impact exhibited emotional and behavioural shifts, such as growing indifference to students’ issues. At the same time, participants described numerous positive impacts of their work, including emotions as sources of empathy, motivation, as well as their work providing them with a sense of purpose and meaning. Rather than interpreting these as contradictory, findings suggest that positives and negatives are intertwined. The same emotional processes that threaten wellbeing can also enhance research work. If researchers were better supported in their work, these positives could be built on while the negative impacts reduced. Given these insights, our study emphasises the need for a structured strategy to prevent, protect against, and address negative impacts in research, enabling the positive potential of emotionally challenging research, and the researchers themselves, to thrive.

### Resources

The Researcher Wellbeing Project resources that were developed by the team specifically to help researchers cope with the impacts discussed in this article can be accessed on our webpage. This includes free institutional guidance, wellbeing plan templates and guidance, risk assessment guidance, what to do if a researcher or participant gets distressed, what to include in a funding proposal. See: https://www.bath.ac.uk/projects/the-researcher-wellbeing-project-rwp-addressing-researcher-distress-trauma-and-secondary-trauma/.

## Data Availability

To maintain the confidentiality and anonymity of the research respondents the data is not publicly accessible.

## Appendix A

### Interview Topic Guide

#### Section 1: Research Topic

On the questionnaire, you indicated that you do research on XXX. Can you tell me a little about that research please and your involvement?

#### Section 2: Experiences of Undertaking this Research

1. How have you found it working in this research area?
  - How does, if at all, this research impact on you?
  b. Does your research link in any way to your own experience? If yes, does this have any additional impact on you?
  c. In what way and at what stage might the research topic, if at all, be distressing for you (and/or your research team/students)? Literature, interviewing, presenting
  d. Are there, or have there been, any emotional or other consequences (mental health consequences) for you of working in this research area? you)?
  e. Have you experienced any mental health consequences as a result of conducting research in this area?
  f. Have you ever felt numb towards your participants?
  g. What have you found interesting or beneficial from undertaking the research?
  h. What have been the things you have found hardest about work in this research area?
  i. Are there particular stages of the research process that you (and/or your team) have found distressing? If yes, please tell us about them.
  j. Was there anything else going on in your life at the time that made it easier or harder for you researching this topic? What influence did that have on you?
2. Have you ever considered changing the research topic because you find it distressing?
  - If yes, please tell us about that and if it was successful.

#### Section 3: Support

1. Is there anything that makes you (or your team) more or less able to cope with the emotionally challenging aspects of your research?
2. Have you ever wanted, asked for or received support with any impacts of undertaking emotionally challenging research? If yes, what and what was that like?
3. Do you have any particular plan or coping strategies to help you cope with the impacts of your research? If yes, what is this?
4. Have you used any formal or informal (or other) support to help you cope with the impacts of your research? If yes, what are these, were they helpful (if so how)?
5. How do you feel about the university? For example, would you turn to them if you had mental health difficulties linked to your research? If yes, can you tell me why? If no, can you tell me why?
6. Can you tell me about the research culture of the university, in terms of whether it is ok to talk about mental health and wellbeing in relation to your research?
7. Are their particular emotion or feeling rules in relation to research at the university? If so what are they? If so how are they communicated to you? Are these applicable to academia more generally?
8. Are there particular feeling rules that we should abide by when doing potentially distressing research (e.g. are there dos and don'ts as a researcher)
9. What do you think about the universities mental health support services?
10.  Was a formal risk assessment done in relation to this research? If so, by whom, and what did it say?
11.  In an ideal world, what would support look like for researchers like you who are working in emotionally challenging research areas?

## Footnotes

1 The World Health Organisation (2012, p. 6) does not recommend psychological debriefing “for people exposed recently to a traumatic event,” as it may exacerbate negative impacts.
